# Brain CB_2_ Receptors: Implications for Neuropsychiatric Disorders

**DOI:** 10.3390/ph3082517

**Published:** 2010-08-10

**Authors:** Michelle Roche, David P Finn

**Affiliations:** 1 Physiology, School of Medicine, NCBES Neuroscience Cluster and Centre for Pain Research, National University of Ireland, Galway, University Road, Galway, Ireland; 2 Pharmacology and Therapeutics, School of Medicine, NCBES Neuroscience Cluster and Centre for Pain Research, National University of Ireland, Galway, University Road, Galway, Ireland; E-Mail: David.finn@nuigalway.ie (D.P.F.)

**Keywords:** endocannabinoid, CB(2), depression, stress, schizophrenia, neurophysiology

## Abstract

Although previously thought of as the peripheral cannabinoid receptor, it is now accepted that the CB_2_ receptor is expressed in the central nervous system on microglia, astrocytes and subpopulations of neurons. Expression of the CB_2_ receptor in the brain is significantly lower than that of the CB_1_ receptor. Conflicting findings have been reported on the neurological effects of pharmacological agents targeting the CB_2_ receptor under normal conditions. Under inflammatory conditions, CB_2_ receptor expression in the brain is enhanced and CB_2_ receptor agonists exhibit potent anti-inflammatory effects. These findings have prompted research into the CB_2_ receptor as a possible target for the treatment of neuroinflammatory and neurodegenerative disorders. Neuroinflammatory alterations are also associated with neuropsychiatric disorders and polymorphisms in the CB_2_ gene have been reported in depression, eating disorders and schizophrenia. This review will examine the evidence to date for a role of brain CB_2_ receptors in neuropsychiatric disorders.

## 1. Introduction

The endogenous cannabinoid (endocannabinoid) system is an important lipid signalling system playing a key role in mediating and modulating physiological responses including central nervous, immune, endocrine, reproductive and cardiovascular system activity. The endocannabinoid system comprises the naturally occurring endogenous ligands (endocannabinoids), the best characterized of which are anandamide (AEA) and 2-arachidonyl glycerol (2-AG); the enzymes involved in the synthesis and degradation of these lipid mediators; and the G-protein coupled cannabinoid receptors CB_1_ and CB_2_, through which the endocannabinoids mediate their effects. Endocannabinoids are synthesized on demand, with the phospholipase D catalysed hydrolysis of *N-*acylphosphatidylethanolamine being primarily responsible for the synthesis of anandamide [1], while 2-AG synthesis is catalysed by the enzymes phospholipase C and diacylglycerol lipase [2]. The enzyme fatty acid amide hydrolyase (FAAH) preferentially catabolises anandamide and although 2-AG also acts as a substrate for FAAH, monoacylglycerol lipase (MAGL) is considered the primary enzyme involved in 2-AG inactivation [3]. In addition to CB_1_ and CB_2_ receptors_,_ endocannabinoids also have affinity for, and activity at, transient receptor potential vanilloid 1 (TRPV1), peroxisome proliferator-activated receptors (PPARs) and GPR55 [4,5,6,7]. Elements of this novel signaling system are widely and densely expressed in the mammalian brain [8,9], implying crucial roles in central nervous system function.

The cannabinoid CB_1_ receptor has been well characterised and is thought to mediate the majority of the psychoactive effects of cannabinoids due to its high level of expression in key central nervous system regions involved in movement, affective responding, cognition, temperature, appetite and neuroendocrine function [10,11,12]. Its localisation is predominantly presynaptic, and its direct activation by synthetic agonists, or by endocannabinoids that signal retrogradely, inhibits the release of neurotransmitters including GABA and glutamate [13]. However, the clinical utility of cannabinoids acting at CB_1_ receptors is limited due to confounding adverse central side effects and the development of tolerance [14,15]. This has led to increased interest in the possible clinical utility of endocannabinoid modulators and selective CB_2_ receptor ligands. The CB_2_ receptor has been categorised classically as the peripheral cannabinoid receptor due to its presence on the cells and tissues of the immune, reproductive, cardiovascular, gastrointestinal and respiratory systems and numerous reports which were unable to detect CB_2_ receptor transcripts in normal healthy brain [12,16,17,18,19]. However, recent evidence, reviewed in detail in Section 2 below, suggests that CB_2_ receptors are present in the brain under normal and, in particular, under pathological conditions, although to a much lesser extent than the ubiquitously expressed CB_1_ receptors. 

The CB_2_ receptor is encoded by a gene located on chromosome 1p36, and was first identified and cloned in 1993 [12]. It shares 48% homology with the CB_1_ receptor, and there is 82% sequence identity between the mouse and human CB_2_ receptor [20]. CB_1_ and CB_2_ cannabinoid receptors are both seven-transmembrane domain receptors coupled to G_i/o_ proteins, activation of which inhibits adenylyl cyclase activity and initiates mitogen-activated protein kinase (MAPK) and phosphoinositide 3-kinase (PI3K)-Akt pathways [20]. The kinases in the MAPK signal transduction pathways activated by CB_2_ receptors include Jun *N*-terminal protein kinase (JNK), extracellular signal-regulated kinase (ERK)1/2 and p38. CB_2_ receptor-mediated inhibition of cAMP and activation of MAPK and PI3K-Akt signaling has been demonstrated in several cell types including those involved in central nervous system function such as microglial cells [21,22,23,24], neural progenitor cells [25,26] and cerebromicrovascular endothelial cells [27]. In addition, CB_2_ receptor activation enhances the synthesis of ceramide, a sphingolipid messenger, particularly in tumour cell lines including human leukemia cell line [28], prostate cancer PC3 cells [29] and DLD-1 and HT29 colon cancer cells [30] and also in neuroglioma [31,32] and human astrocytoma cells [33], a mechanism which induces apoptotic cell death. Based on the ability to inhibit forskolin-stimulated cAMP accumulation, it has been demonstrated that anandamide has low intrinsic activity at the CB_2_ receptor while 2-AG acts as a full agonist [34,35,36,37] and potentially the true natural agonist at the CB_2_ receptor. However, under pathological conditions anandamide levels are enhanced and have been proposed to mediate significant effects via CB_2_ receptors [22,38].

Due to the high density of CB_2_ receptors on peripheral tissues, particularly in cells and tissues of the immune system, the anti-inflammatory and antinociceptive effects of CB_2_ receptor activation have received a great deal of attention. This research has led to the development of an ever increasing number of selective agonists for the CB_2_ receptor [39] some of which have undergone clinical evaluation. For example, Pharmos Corporation demonstrated that Cannabinor (formally PRS-211,375) did not elicit significant analgesic effects on capsaicin-induced pain but did so on pressure-induced and heat-induced pain and was well tolerated by patients in Phase 2 of clinical trials [40]. However according to their website [40] Pharmos Corporation has since decided to cease the development of Cannabinor for pain indications and explore possible collaborations towards retargeting this CB_2_ receptor agonist. Similarly, GlaxoSmithKline have also completed Phase 2 clinical trials evaluating the effects of the selective CB_2_ agonist GW842166, in osteoarthritis [41] and dental pain [42], although the results of these studies have not been published to date. Identification of CB_2_ receptors in the central nervous system has also led to increasing investigation into its involvement in neuroimmunological and neurodegeneratative disorders, topics which have been covered in detail by several recent reviews [43,44,45,46,47,48,49,50]. The present review will provide an overview of the evidence demonstrating the presence of functional CB_2_ receptors in the brain, the role of this receptor in neurophysiology and highlight the potential involvement of these receptors in neuropsychiatric disorders.

## 2. Expression and Distribution of CB_2_ Receptors in the Central Nervous System

Several researchers have failed to identify CB_2_ receptor mRNA or protein in the brain under non-pathological conditions [12,16,17,18,19]. However, with the development of increasingly selective and sensitive tools for identifying CB_2_ receptors, there is now evidence demonstrating low expression of this receptor in the brain, although the functional significance of this expression remains to be fully elucidated. CB_2_ receptors have been identified on human cerebral microvascular endothelial cells [27], human foetal astrocytes [51] and limited populations of microglia [52,53] in the healthy human brain. A recent study employing *in situ* hybridization using specific riboprobes demonstrated CB_2_ receptor transcripts within the cerebral cortex, hippocampus and the globus pallidus of adult male *Macaca fascicularis* primate [54]. Comparably, CB_2_ receptor expression has been demonstrated in the cerebral cortex, hippocampus, striatum, amygdala, thalamic nuclei, periaquaductal grey, cerebellum and several brain stem nuclei of the rodent brain [55,56,57,58,59,60,61]. Although many studies have identified central CB_2_ receptors on glial and endothelial cells, there is mounting evidence to support the expression of CB_2_ receptors on sub-populations of neurons within the central nervous system. *In vitro* studies have demonstrated the presence of CB_2_ receptor mRNA and/or protein on human sensory nerve fibres [62], dorsal root ganglia and spinal cord neurons [63,64], hippocampal neuronal cultures [25,26,56] and cerebellar Purkinje neurons [63]. 

One of the first studies to demonstrate CB_2_ receptor expression on neurons *in situ* in the brain was that of Skaper *et al.*, who reported expression on cerebellar granular cells in the neonatal mouse brain [65]. At the time, research suggested that CB_2_ receptors were absent from the brain and therefore the authors concluded that agonism of a CB_2_-like receptor downregulates excitotoxic neuronal injury. The presence of CB_2_ receptors on neurons, microglia and capillary endothelia, but not astrocytes, in the cerebellum was subsequently confirmed in several studies [56,57,58,60,66]. However, the precise location and distribution of CB_2_ receptors on cerebellar neurons remains to be fully characterised. Several studies have demonstrated an association between the CB_2_ receptor and Purkinje cell bodies and dendrites [56,57,60], however a recent study failed to confirm this association [58]. This latter study identified CB_2_ receptors on cluster basket cell axons and parallel fibres in the molecular layer which may be associated with granular cells or mossy fibres in the granular layer. Similarly, CB_2_ receptors were originally postulated to be located post-synaptically on the cerebellar Purkinje dendrites [56], however, recent evidence suggests that both CB_1_ and CB_2_ receptors in the cerebellum are principally located pre-synaptically [58], suggesting that they, like CB_1_ receptors, may also play a role in endocannabinoid retrograde neurotransmission. 

CB_2_ receptors have also been localised on neurons in various rodent brainstem nuclei including the dorsal motor nucleus of the vagus, the nucleus ambiguous, spinal trigeminal nucleus [59], vestibular and cochlear nuclei [56,58,67] and inferior olive [58]. The dorsal motor nucleus of the vagus is the integration site of emetic reflexes and co-activation of CB_1_ and CB_2_ receptors on neurons in this region induces anti-emetic effects in the ferret [59]. This was one of the first studies to highlight that although CB_2_ receptor expression in the brain is low under normal non-pathological conditions, its presence on brainstem neurons is of functional significance. 

Expression of CB_2_ receptors outside the cerebellum and brainstem under non-pathological conditions has been controversial. Several investigators have failed to identify CB_2_ receptor transcripts or protein in forebrain regions [12,16,17,18,19]. However, Gong *et al.* provided the first evidence indicating that CB_2_ receptors may have a wider distribution in the brain when they demonstrated (using immunohistochemistry) CB_2_ receptor expression on both neuronal and glial processes in various rat brain regions including the cerebral cortex, hippocampus, striatum, amygdala, thalamic nuclei, periaquaductal grey, cerebellum and several brain stem nuclei [56,60]. However, in the same study [56], CB_2_ receptor mRNA expression was only identified in the striatum and hypothalamus and not in the olfactory bulb, cortex, thalamus or spinal cord. Further evidence for the expression of CB_2_ receptors in cortical areas includes reports of a small proportion of CB_2_ receptors identified on neocortical neurons [68] and moderate to heavy immunolabelling of dendrites and cell bodies of pyramidal neurons in the rat and mouse cerebral cortex [69]. In addition, recent evidence demonstrated CB_2_ expression on pyramidal neurons within layers III and V of the primate cerebral cortex [54]. CB_2_ receptors have also been identified on neural progenitor cells of the subgranular zone of the dentate gyrus in the hippocampus [25] and interneurons primarily in CA1 and CA3 areas of the primate and rodent hippocampus [54,56,70,71]. The expression pattern of CB_2_ receptors in the hippocampus appears somewhat at odds between that reported for pre-pubertal [70] and adult [56] rats suggesting that CB_2_ receptor expression may change as a consequence of development. Evidence suggests that CB_2_ receptors are located primarily in cell bodies and dendrites, but not axons, in cortical areas and the hippocampus [55,60,70,71], indicating a post-synaptic localisation of these receptors. In comparison, both small unmyelinated axons and small dendrites in the substantia nigra exhibit CB_2_ receptor immunoreactivity, suggesting both pre- and post-synaptic localisation in this region [55]. The specific type of neurons expressing CB_2_ receptors and the functional significance of pre- and post-synaptic CB_2_ receptors remain to be determined. Recent evidence suggests that CB_2_ receptors may modulate GABAergic neurotransmission, at least in the entorhinal cortex [72]. In this study, CB_2_ receptor agonism with JWH-133 or 2-AG resulted in suppression of GABAergic inhibition in the medial entorhinal cortex while addition of the CB_2_ receptor antagonist/inverse agonist JTE-907 alone enhanced GABAergic transmission in this region.

Liu and colleagues recently identified two different isoforms of the CB_2_ receptor gene, the expression of which are species- and tissue-specific [73]. In this study, a new isoform of the human CB_2_ gene was identified, CB_2_ gene promoter transcribing testis (CB_2A_), which has a starting exon located 45kb upstream of the previously identified isoform from the spleen (CB_2B_). The authors demonstrate that CB_2A_ mRNA expression is highest in the human testis, and to a lesser extent (<1% of testis expression) in the brain, when compared to the CB_2B_ isoform which is expressed predominantly in the spleen, with very low levels (<0.1% of spleen expression) observed in the brain. CB_2A_ mRNA expression was observed in the human amygdala, caudate, putamen, nucleus accumbens, hippocampus, cortex and cerebellum. It is possible that the failure of previous studies to demonstrate CB_2_ mRNA expression in the human brain may be due to the use of primers targeting the CB_2B_ isoform rather than the CB_2A_ isoform which appears to be more abundant in the brain. Identification of the different isoforms of the CB_2_ receptor has important implications for the development of agents targeting central receptor isoforms that may be devoid of associated widespread anti-inflammatory effects and *vice-versa*. In comparison to the human, mouse and rat homologues of both the CB_2A_ and CB_2B_ isoforms were identified in the spleen and in brain regions including the frontal cortex, striatum and brainstem (1% of the expression levels in the spleen). Expression of CB_2A_ was approximately 5-fold greater than CB_2B_ in the rodent brain [73]. This further highlights the difference between the human and rodent CB_2_ receptor genes and has important implications for the interpretation of results across species. Deletion of the C-terminus of the CB_2_ receptor is the most common means of generating the CB_2_ receptor knockout mouse [74]. Lui *et al.* demonstrated that in *C*-terminus CB_2_ receptor knockout mice, CB_2A_ expression is downregulated and CB_2B_ expression is enhanced in the spleen. In comparison, both CB_2A_ and CB_2B_ promoter activity and expression are enhanced in the brainstem of these knockout mice [73]. The authors suggest that enhanced levels of the CB_2A_ and CB_2B_ isoforms may reflect enhanced expression of a truncated CB_2_ receptor in the brain which may still be functionally active. Although further confirmatory studies are required, these findings may have important implications for the interpretation of results from studies using these mice. 

The high expression of CB_2_ receptors on all components of the immune system and the potent anti-inflammatory effects of CB_2_ receptor agonists has lead to increased interest in the involvement of CB_2_ receptors in immune responses in the CNS. The first evidence for the possible involvement of brain CB_2_ receptors in neuroinflammation was provided by Benito and colleagues, who demonstrated the presence of CB_2_ receptors on microglia associated with neuritic plaques in Alzheimer’s disease [52]. CB_2_ expression has since been demonstrated in several other pathologies including adult and paediatric brain tumours [75], multiple sclerosis [76,77], amyotrophic lateral sclerosis [77], Down’s syndrome [78], Huntington’s disease [79] and HIV-induced encephalitis [80]. The involvement of CB_2_ receptors in the brain in these pathologies is further supported by preclinical studies demonstrating enhanced expression in the experimental autoimmune encephalomyelitis (EAE) model of multiple sclerosis [81,82], ischemia-reperfusion injury [83,84], R6/2 transgenic mouse model of Huntington’s disease [79], transgenic model of amyotrophic lateral sclerosis [85] and in response to simian immunodeficiency virus encephalitis [80] and lipopolysaccharide injection [86]. Therefore, neuroinflammation is associated with enhanced CB_2_ receptor expression in the brain while CB_1_ receptor expression may be unaltered [52], reduced [87,88,89] or increased [82,90] under these conditions. 

In addition to neuroinflammatory disorders, drugs of abuse and other pharmacological agents also modulate brain CB_2_ receptor expression. Enhanced CB_2_ receptor mRNA expression in whole brain extracts have been demonstrated following chronic administration of cocaine and heroin [91]. In addition, Torres and colleagues recently demonstrated enhanced CB_2_ receptor immunoreactivity in the frontal cortex of rats 3 hours following MDMA administration, an effect attenuated by repeated administration of the CB_2_ receptor agonist JWH-015 [92]. In contrast, preference for alcohol consumption was associated with a reduction in CB_2_ receptor transcripts in the ventral midbrain and striatum [69,93]. Chronic administration of the non-selective CB_1_/CB_2_ receptor agonist WIN55,212-2 significantly enhances CB_2_ receptor expression in the cerebellum but not frontal cortex, hippocampus, striatum, spleen or testis [73]. There was no effect of repeated administration of CB_1_ (AM251) or CB_2_ (AM630) antagonists on CB_2_ expression in the mouse brain [73]. 

## 3. Neurophysiological Functions Mediated by Central CB_2_ Receptors

Several studies have reported that CB_2_ receptor agonists/antagonists are devoid of psychoactive effects and have attributed the CNS effects of CB_2_ ligands to non-selective activity at CB_1_ or other non-cannabinoid receptors [94]. However, with increasing evidence for the presence of CB_2_ receptors in the brain, particularly on sub-populations of neurons, the involvement of this receptor in mediating possible neurological and psychoactive effects is receiving increasing attention. Alterations in locomotor responses and stereotyped behaviour observed in mice following the administration of high doses of selective CB_2_ receptor agonists/antagonists provides some evidence for a neurophysiological role of brain CB_2_ receptors, although non-selective effects at these doses cannot be ruled out. Acute blockade of the CB_2_ receptor using the antagonist SR144528 induces biphasic effects, increasing spontaneous locomotor activity in the DBA/2 mouse at low doses (1-10mg/kg) and reducing activity at high doses (20 mg/kg) [91]. In comparison, genetic deletion of the CB_2_ receptor is not associated with any change in motor co-ordination [95,96]. Increasing doses of the CB_2_ receptor agonist JWH-015 reduced locomotor activity and stereotyped behaviour, with females of three different mouse strains more affected than their male counterparts [60]. However, the authors did not determine if these effects were mediated specifically by CB_2_ receptor activation or due to effects at other non-selective targets. Similar locomotor depressant effects were observed following administration of an alternative CB_2_ receptor agonist GW405833 (100 mg/kg) [97], however a follow-up study determined that the central effects of GW405833 were not mediated by CB_2_ receptors, determined using CB_2_-/- mice [95]. In addition, the selective CB_2_ receptor agonists HU308 and AM1214, at doses that induced appreciable antinociceptive effects, did not affect locomotor activity [98,99]. Recent evidence has also demonstrated that administration of the selective CB_2_ receptor agonist AM1241, or antagonist AM630, failed to elicit a change in brain activity assessed by fMRI [100], leading these authors to conclude that CB_2_ receptors in the brain may not be functionally active under non-pathological conditions. However, it is also possible that the extent to which the CB_2_ receptor was modulated pharmacologically in this study may not have been of sufficient magnitude to elicit changes in neuronal activity detectable with fMRI methodology. 

It has been proposed that functional interaction or co-operation between CB_1_ and CB_2_ receptors is required in order regulate neurophysiological function. This would account for the low intrinsic activity of CB_2_ ligands, administered alone, on CNS function. In line with this theory, Van Sickle and colleagues demonstrated that co-activation of both CB_1_ and CB_2_ receptors on neurons in the dorsal motor nucleus of the vagus in the brainstem reduces emetic responses to morphine-6-glucuronide in the ferret [59]. However, CB_2_ receptor activation on dorsal motor nucleus of the vagus neurons using the selective agonist AM1241, or the endocannabinoid 2-AG, was not sufficient to inhibit emetic reflexes in the absence of CB_1_ receptor stimulation. Thus, activation of brain CB_2_ receptors alone may not be sufficient to modulate significant physiological effects such as emesis under non-pathological conditions.

It is well known that CB_1_ receptors are involved in the regulation of food intake and energy expenditure, prompting several companies to develop CB_1_ receptor antagonists/inverse agonists such as rimonabant, as potential anti-obesity agents [101]. However, repeated administration of these agents was associated with adverse psychiatric effects such as anxiety and depression in a small number of individuals, resulting in the withdrawal of the rimonabant from the European market. As such, attention has now turned to the involvement of endocannabinoids and CB_2_ receptors in regulating energy balance. Eating disorders such as anorexia nervosa and bulimia nervosa have recently been associated with a Q63R polymorphism of the CB_2_ gene, with the R allele significantly more abundant in these individuals than controls [102]. Peripheral administration of the CB_2_ receptor partial agonist palmitoylethanolamide (PEA), and the CB_2_ receptor antagonist AM630, decreased food intake in non-fasting C57Bl/6 and DBA/2 mice [103]. The appetite suppressant effects of PEA were not affected by 12 hour food deprivation. However, following food deprivation, AM630 increased food intake in C57Bl/6 mice [103]. Similarly, intracerebroventricular administration of AM630 (5 µg) increased deprivation-induced food intake in male Lewis rats although no effect was observed at higher doses [104]. Thus, while antagonism of the CB_1_ receptor induces anorexia irrespective of fasting state, the effects of CB_2_ receptor ligands on food intake appear to depend on metabolic state. Thus, both CB_1_ and CB_2_ receptors in the brain appear to influence food intake in rodents although the precise mechanisms by which this occurs remain to be determined.

CB_2_ receptor agonists induce potent anti-hyperalgesic effects in models of acute, neuropathic and inflammatory pain [for reviews see [105,106,107]] although the involvement of central CB_2_ receptors in this response has not received a great deal of attention. One of the few studies to demonstrate an involvement of brain CB_2_ receptors in modulating nociceptive responding reported a reduction of noxious and non-noxious evoked responses in a rat model of neuropathic pain following the administration of the CB_2_ receptor agonist JWH-133 into the ventral posterior nucleus of the thalamus [108]. Similarly, intrathecal but not peripheral administration of JWH-133 reversed mechanical allodynia associated with peripheral nerve injury in mice [109], further highlighting the involvement of central CB_2_ receptors in mediating neuropathic pain. The molecular mechanism mediating central CB_2_-induced anti-hyperalgesic effects is unknown, however, evidence indicates a functional interaction between CB_2_ and µ-opioid receptors, with SR144528 inhibition of CB_2_ receptors attenuating a noladin ether-induced decrease in µ-opioid receptor activity in the forebrain [110] while SR144528 alone reduces µ-opioid receptor activity and expression in the brainstem and cerebellum [111,112]. It is also possible that CB_2_ receptor agonists mediate their analgesic effects by inhibition of neuroinflammatory activity associated with neuropathic pain. Recent evidence has demonstrated a slight but non-significant increase in CB_2_ receptor expression in the spinal cord of a rat model of diabetic neuropathic pain [113], however it remains to be determined if central expression of this receptor is altered in other neuropathic pain states. It should also be noted that intra-thalamic administration of JWH-133 or SR144528 does not alter nociceptive responding in sham-controls [108]. Similarly, intracerebroventricular administration of JWH-133 did not alter inflammatory nociceptive responding following intra-plantar formalin administration [114] and intra-amygdaloid administration of the non-selective CB_1_/CB_2_ receptor agonist WIN55-212,2 attenuated formalin-induced nociceptive behaviour in rats via activity at CB_1_ but not CB_2_ receptors [115]. These findings further indicate that the physiological effects of brain CB_2_ receptors on nociceptive processing under non-pathological conditions may be minimal, but these receptors do appear to play a role under conditions of neuropathic pain. 

## 4. Neuroinflammation and CB_2_ Receptors

Neuroinflammation encompasses a wide array of cellular processes including activation of microglia and astrocytes, enhanced pro-inflammatory cytokines, chemokines, eicosanoids, complement activation and acute phase proteins. Cannabinoid compounds, plant-derived, synthetic and endogenous, are well known to elicit potent effects on inflammation, both peripherally and centrally. The presence of CB_2_ receptors on glia and neurons in the brain, has prompted several groups to investigate the role of this receptor in neuroinflammation and neuroprotection, a topic which has been covered in detail by several recent reviews [43,44,45,46,47,48,49]. This body of evidence suggests that CB_2_ receptor activation elicits glial-dependant anti-inflammatory effects, thereby reducing neuroinflammation associated with several neurodegenerative diseases. Neuroinflammatory processes have also been proposed to underlie the pathophysiology of several neuropsychiatric disorders and for this reason a brief overview of the role of CB_2_ receptors in neuroinflammation and its implications for psychiatric disorders will be presented here.

Enhanced CB_2_ receptor expression, primarily on activated microglia, occurs in several neurodegenerative disorders including Alzheimer’s disease, multiple sclerosis and Huntington’s disease, and in experimental models of neuroinflammation [75,78,79,80,81,82,83,84,86]. Selective CB_2_ receptor agonists reduce symptoms and slow the progression of neurodegeneration in an animal model of amyotrophic lateral sclerosis [85] and in the EAE model of multiple sclerosis [82]. In turn, CB_2_ receptor deletion is associated with exacerbated neuroinflammatory responses and symptomatology in several animal models including EAE [82] and cerebral ischemic/reperfusion injury [116]. Expression of CB_2_ receptors on microglia alters depending on their state of activation, with little to no receptors observed on microglia in the healthy brain [24,44,117,118]. Upregulation of CB_2_ receptors on microglia, as occurs in response to inflammatory conditions, modulates the activation, proliferation, differentiation and migration of these cells [21,24,119]. It is widely accepted that CB_2_ receptor activation is associated with potent anti-inflammatory responses, including inhibition of the release of inflammatory mediators including nitric oxide and cytokines such as interleukin (IL)-1, tumour necrosis factor (TNF)-α and IL-6 from both rodent and human microglial [22,23,120,121] and astrocytic [51,122,123] cells and enhanced release of anti-inflammatory cytokines such as IL-10 and IL-1 receptor antagonist (IL-1ra) [122,124]. *In vivo* studies have demonstrated that stimulation of CB_2_ receptors reduces microglial activation and the expression of pro-inflammatory cytokines in models of neuroinflammation [119,125], hypoxia-ischemia [90] and Huntington’s disease [79,126]. In the case of Alzheimer’s disease, pro-inflammatory cytokines in neuritic plaques interfere with the ability of microglia to phagocytose Aβ [127]. CB_2_ receptor activation is associated with enhanced removal of Aβ by THP-1 derived macrophages in Alzheimer’s disease brain sections [128]. Thus, it has been proposed that the increased ability of microglial cells to phagocytose Aβ is a consequence of the anti-inflammatory effects of CB_2_ receptor activation. Overall, the upregulation of CB_2_ receptors observed in neuroinflammatory and neurodegenerative disorders possibly acts as a regulatory mechanism controlling the production and release of toxic inflammatory mediators from microglia and/or enhancing the neuroprotective effects of astrocytes. 

CB_2_ receptors also regulate neuronal proliferation and survival [25,26,66,129]. CB_2_ receptor deficient mice exhibit reduced neural progenitor proliferation [25] and CB_2_ receptor antagonism is associated with a reduction in the development of new neurons in the olfactory bulb [129]. In comparison, selective agonism of the CB_2_ receptor results in enhanced neural stem cell proliferation, possibly via stimulation of MAPK-ERK and Akt pathways [25,26,129]. Increasing endocannabinoid tone using a reuptake inhibitor protects against AMPA-induced excitotoxicity, via modulation of CB_1_, CB_2_ and PPARγ signalling [130] and CB_2_ receptor agonism protects the striatum from malonate-induced neurotoxicity [126]. Furthermore, chronic administration of the non-selective CB_1_/CB_2_ agonist HU210 enhances neurogenesis in adult mice [131], and WIN55,212-2 triggers neurogenesis in the hippocampus of aged rats [132], effects which may, in part, be mediated by activity at CB_2_ receptors. Although these findings have important implications for the development of novel therapeutics for acute brain injury and chronic neurodegenerative disorders, it is possible that CB_2_ receptors may also have a role to play in psychiatric disorders such as depression and schizophrenia which are associated with enhanced inflammatory responses and reduced neurogenesis. 

## 5. CB_2_ Receptors and Neuropsychiatric Disorders

### 5.1. Stress and Anxiety

The role of the endocannabinoid system in mediating fear, stress and anxiety has been researched extensively over the past decade (for recent reviews see [133,134,135]). In general, CB_1_ receptor activation elicits complex bi-phasic effects on stress-responding and recent evidence indicates that this may in part be due to differential activation of CB_1_ on forebrain glutamatergic and GABAergic neurons that elicit anxiolytic and anxiogenic effects respectively [136]. As mentioned in earlier sections, several studies have demonstrated a lack of psychoactive effects such as catalepsy, hypolocomotion and hypothermia following pharmacological modulation of the CB_2_ receptor [98,99,137] and, as a consequence, the role of CB_2_ receptors in regulating stress and anxiety has received little attention. The data reviewed above in Section 2 demonstrating expression of CB_2_ receptors in key brain areas involved in modulating the stress response including the amygdala, hippocampus, prefrontal cortex and hypothalamus [56,60,70,71,138], suggest that the potential role of CB_2_ receptors in regulation of emotional responding is at least worthy of investigation. To date, most studies examining the effect of selective CB_2_ receptor agonists on CNS function have examined the ability of ligands to modulate locomotor activity, with few studies examining effects in validated models of emotionality/anxiety. Unconditioned responding in stressful environments or conditioned responses to a previously learned aversive stimulus are the most commonly used means of assessing anxiety-related behaviour in animals. Onaivi and colleagues have assessed stress-induced anxiety-related behaviour in the two-compartment black and white test (also known as the light-dark test) and in the elevated plus maze following the administration of CB_2_ receptor ligands [60,91]. Acute systemic administration of JWH-015 (1-20 mg/kg) dose dependantly induced an anxiogenic response in the black and white box, with females slightly more sensitive than males [60,139]. In contrast, JWH-015 (20 mg/kg) attenuated stress-induced gender-specific aversion to the open arms of the elevated plus maze [139] and administration of the CB_2_ receptor agonist GW405833 (100 mg/kg) induced anxiolytic effects in the marble burying test [97]. However, it should be noted that the behavioural effects of CB_2_ receptor stimulation in these studies was accompanied by reduced locomotor activity at the doses used [60,97,139], and administration of a CB_2_ receptor antagonist failed to reverse either the locomotor depressant or the anxiolytic effects observed in the marble burying test [95], which may have important implications for the interpretation of the effects observed. Chronic administration of JWH-015 results in an anxiolytic behavioural profile in the black and white box [60] and reduces stereotypic behaviour in non-stressed but not stressed BALB/c mice [139], which the authors interpret as an anxiolytic profile. However, it should be noted that stereotypic behaviour represents locomotor activity and rearing and, as such, may be more accurately interpreted as a measure of general activity rather than a measure of anxiety-like behaviour. 

In contrast to the effects observed following CB_2_ receptor stimulation, CB_2_ receptor antagonism using SR144528 had little or no effect in the black and white box, with the exception of a decrease in time spent in the white chamber in DBA/2 male mice at 20 mg/kg SR144528 [91], again possibly a result of reduced locomotor activity observed at this dose. Repeated (3 day) intracerebroventricular administration of CB_2_ antisense oligonucleotide increased the amount of time spent on the open arms of the elevated plus maze indicative of an anxiolytic-like effect [61,139], however, the effect of this treatment on locomotor activity in the maze was not reported. Only one published study to date has examined the role of brain CB_2_ receptors in conditioned aversion/learning, demonstrating that infusion of JWH-015 or PEA into the CA1 region of the hippocampus does not affect novel object recognition or long-term memory retention [140]. Overall, the results obtained from behavioural studies of the role of CB_2_ receptors in modulating the response to aversion are far from clear, highlighting the need for further studies examining the effects of selective deletion, blockade or stimulation of brain CB_2_ receptors on the regulation of emotional responding.

A critical component of the stress response is the activation of the hypothalamic-pituitary-adrenal (HPA) axis and the subsequent increase in glucocorticoid levels and several studies have examined the role of the endocannabinoid system in mediating this response [141]. CB_2_ receptor mRNA and protein have been identified in the brain regions that modulate HPA axis activity including the hippocampus, amygdala and hypothalamus [56,71], while in comparison, CB_2_ receptors are not expressed in the adrenal cortex [142], indicating that should CB_2_ ligands modulate the neuroendocrine response to stress, this would most likely occur at the level of the brain. Our studies have indicated that endotoxin-induced increases in circulating corticosterone levels are not altered by administration of the non-selective CB_1/2_ receptor agonist HU-210 or the CB_2_ receptor antagonist/inverse agonist SR144528 [125]. Further studies will be required to determine whether CB_2_ receptors are involved in the endocannabinoid-mediated modulation of neuroendocrine activity under basal and stress conditions. 

Although Onaivi and colleagues demonstrated similar effects of the CB_2_ receptor ligand JWH-015 on locomotor activity and stereotypic behaviour in three strains of mice, namely C57Bl6/J, DBA/2 and BABL/c, they did not compare behavioural responses between these different strains in tests of emotionality and anxiety [139]. BALB/c mice have been proposed as a model of anxiety and are regarded as more stress-sensitive than other mouse strains including C57Bl6/J and DBA/2 mice [143,144,145]. For example, BALB/c mice exhibit an anxiogenic profile in the black and white box, manifested as a reduced time in the white compartment of the test apparatus when compared to C57Bl6/J counterparts [145]. Therefore, due to the contribution of genetic background to stress responding, it is possible that CB_2_ ligands may elicit differential effects depending on the strain of mouse used. In accordance, low doses of JWH-015 (5 mg/kg) increased stereotypic behaviour in male DBA/2 but not C57Bl6/J or BALB/C mice [60]. No significant difference in whole brain CB_2_ receptor expression prior to or following chronic stress was observed between the three different mouse strains [139]. However, it remains to be determined if the density or function of CB_2_ receptors or other components of the endocannabinoid system are differentially altered between these different strains of mice in brain regions associated with stress responding. Accordingly, early life stress such as maternal deprivation induces an anxiogenic behavioural phenotype [146] which has recently been associated with enhanced CB_2_ receptor expression in the hippocampus [70]. Thus, greater understanding of the role of CB_2_ receptors in pathological anxiety states will be reached by examining the expression, distribution and functional activity of these receptors in preclinical models that exhibit ethological validity. 

### 5.2. Depression

The involvement of the endocannabinoid system in the regulation of mood and affective responding has received increasing interest in the past few years (for recent reviews see [147,148,149]), particularly in light of the withdrawal of rimonabant (CB_1_ receptor antagonist/inverse agonist) as an anti-obesity agent due to the increased risk of psychiatric side effects, including depression. The involvement of CB_1_ receptors in regulating mood is further supported by the upregulation of CB_1_ receptor expression and function in the prefrontal cortex of depressed suicide victims [150] and enhanced CB_1_ receptor density in the prefrontal cortex of alcoholic suicide victims compared with alcoholic controls [151]. Furthermore, allele variations in the CB_1_ receptor gene *CNR1*, plays a role in the antidepressant response in major depressed patients [152] and a cohort of elderly depressed Parkinsonian patients have demonstrated a polymorphism (AAT*n*) of the *CNR1* [153]. Preclinical evidence further supports the involvement of CB_1_ receptors in depressive-like behaviour [154,155] and suggests that the effects of currently used antidepressants might depend upon endocannabinoid system modification. For example, endocannabinoid-CB_1_ receptor signaling in the brain is altered by several interventions that elicit antidepressant activity in humans, including chronic tricyclic antidepressant treatment [156,157], repeated electroconvulsive therapy [158] and sleep deprivation [159]. In contrast, the involvement of the CB_2_ receptor in affective responding has not received similar attention. A recent study has, however, demonstrated an association between depression and a polymorphism in the CB_2_ receptor gene at position Q63R in Japanese patients [69]. Polymorphisms in the Q63R in the CB_2_ gene have also been linked with eating disorders, alcoholism, osteoporosis, autoimmune disease and schizophrenia [93,102,160,161], many of which often demonstrate co-morbidity with depressive illness. Although it remains to be determined if this genetic link exists across other ethnic groups, it is possible that genetic variation in the CB_2_ gene may be a predisposing factor in the development of depression. CB_2_ receptor mediated effects of cannabinoid agonists such as WIN55,212-2 and 2-AG are reduced in the presence of the Q63R polymorphism [160,162] and serum levels of 2-AG and anandamide are reduced in patients with major depression, an effect directly correlated with the duration of the depressive episode [163]. Although further studies are required, in addition to alterations in endocannabinoid levels and CB_1_ receptor signaling, central CB_2_ receptor expression or function may be altered in depressed patients. 

The association between adverse life events and the development of depression is widely acknowledged, and, as a consequence, many of the preclinical models of depression or antidepressant-like activity exploit this link by examining behavioural and physiological responses to stress. One of the most widely used behavioural tests for antidepressant-like activity is the forced swim test, where rodents exposed to a confined swim arena will initially attempt to escape but after some time will assume a floating posture (immobility) that is thought to be related to a state of behavioural despair [164]. Antidepressant agents increase escape behaviour thereby reducing the duration of immobility, while in comparison, an increase in immobility is regarded as a depressive-like state. Antidepressant-like activity of cannabinoid ligands and endocannabinoid modulators such as FAAH and anandamide reuptake inhibitors, have been demonstrated in the forced swim test [131,155,165,166,167,168,169,170,171]. In many of the studies, the antidepressant-like effects have primarily been attributed to activity at the CB_1_ receptor, confirmed using receptor antagonists and/or genetic deletion. Recent evidence has indicated that CB_1_ receptors on subpopulations of glutamatergic, but not GABAergic, neurons appear to mediate the forced swim stress-induced behavioural and neuroendocrine effects [172]. However, non-selective CB_1_/CB_2_ receptor agonists and endocannabinoid modulators may also activate CB_2_ receptors. Antidepressant-like activity following chronic CB_1_/CB_2_ receptor stimulation using HU210, but not CB_1_ receptor agonism (AM281) alone, was observed in the forced swim test [131]. In addition, intra-hippocampal administration of HU210 induced an antidepressant-like effect in the forced swim test, an effect only partially attenuated by pharmacological blockade of the CB_1_ receptor [169]. Although further studies are required in order to further determine the mechanism of action underpinning these effects of non-selective CB_1_/CB_2_ agonists, it is possible that activation of central CB_2_ receptors may co-operatively augment the effects of CB_1_ receptor activation on stress-induced behaviour and monoaminergic function. Presently, only one study has been published directly assessing the effect of pharmacological CB_2_ agonism in the forced swim test, reporting that acute administration of the CB_2_ agonist GW405833 did not alter time spent immobile in the forced swim test [173]. However, in the presence of neuropathic pain, CB_2_ receptor activation reduced nociceptive responding to a mechanical stimulus while concurrently attenuating the enhanced immobility observed in the forced swim test [173]. The present results indicate that CB_2_ receptor agonism may alleviate neuropathic pain and the co-morbid depressive symptoms that often accompany this disorder. In an interesting study published recently, García-Gutiérrez and colleagues employed both pharmacological and genetic approaches to investigate the role of the CB_2_ receptor in depressive-like behaviour [138]. Transgenic mice engineered to over-express the CB_2_ receptor (including over-expression in key brain regions implicated in depression) exhibited reduced depressive-like behaviour in the tail suspension test and in a novelty suppressed feeding test, compared with wildtype controls. However, acute intraperitoneal administration of the CB_2_ receptor antagonist AM630, at doses that had no effect on locomotor activity, had an antidepressant-like effect in the forced swim test in wildtype mice, but not in CB_2_ over-expressing mice [138]. Though the depression-resistant endophenotype associated with CB_2_ over-expression and the antidepressant-like effects of CB_2_ receptor blockade pose an apparent discrepancy which is difficult to reconcile, these data do at least demonstrate an important role for the CB_2_ receptor in regulating depressive state in mice. More studies examining the effects of direct activation/inhibition of brain CB_2_ receptors are required in order to determine conclusively the role this receptor plays a role in stress-induced behavioural changes.

A detailed understanding of the neurobiological underpinnings of depression requires pre-clinical models that mimic the neurological and physiological alterations that characterise this psychiatric disorder. Chronic mild or unpredictable stress (CMS) is a widely used and validated preclinical model of depression displaying several behavioural and physiological alterations that mimic those observed in the clinical setting [174,175]. CMS is associated with reduced 2-AG levels in the hippocampus, enhanced anandamide levels in limbic and cortical areas and differential expression of CB_1_ receptor with increased receptor expression in the prefrontal cortex and a concurrent decrease in the hippocampus, hypothalamus and ventral striatum [156,176]. One of the first studies to imply that the CB_2_ receptor may be implicated in depression was that of Onaivi *et al.*, where they demonstrated that CB_2_ protein levels measured by western immunoblotting in whole brain extract were enhanced in mice subjected to CMS for a period of 4 weeks [61,69]. However, the anatomical region(s) associated with the enhanced CB_2_ protein expression or the identity of the cells that express this receptor, *i.e.*, glia or neurons, were not identified. CB_2_ receptor mRNA was detected in the striatum, midbrain and hippocampus of control and CMS exposed mice, however, no significant difference in expression was observed between the groups [61]. More recently, García-Gutiérrez and colleagues demonstrated that CMS in mice for 7-8 weeks resulted in reduced levels of CB_2_ mRNA in the hippocampus of mice, compared with non-stressed controls. Moreover, this stress-induced reduction was prevented by chronic administration of the CB_2_ receptor antagonist AM630 [138]. Behaviourally, acute treatment with JWH-015 reduced stereotypic behaviour in stressed but not non-stressed mice whereas the converse was observed following chronic treatment [139]. This group have also reported that reduced spontaneous locomotor activity of mice subjected to CMS was enhanced by acute and chronic treatment with JWH-015. In addition, CMS-induced anxiogenic behaviour in the elevated plus maze was attenuated by acute administration of JWH-015 [60,69]. CMS is associated with reduced intake of palatable solutions such as sucrose, used as a measure of anhedonia, a hallmark of depressive-illness. Daily administration of either the CB_2_ receptor agonist JWH-015 or the CB_2_ receptor antagonist AM630 did not alter chronic stress-induced decreases in sucrose consumption [69,91]. Administration of JWH-015 for a period of 2 weeks increased sucrose consumption in control but not stressed animals, an effect not observed at later time points. Although the authors report no effect of AM630 on sucrose consumption, examination of the data indicates that sucrose consumption was reduced in control, but not stressed mice, from week 2 of treatment with this CB_2_ receptor antagonist (3 mg/kg) [69]. Based on these findings it would appear that stress blocks CB_2_ receptor modulation of hedonic responses. Similarly, although CB_2_ receptor ligands elicit no effect on alcohol consumption in control mice, enhanced alcohol consumption following CMS is augmented by JWH-015 and slightly attenuated by AM630 [93]. In addition to providing further evidence for a differential role of CB_2_ receptors in modulating behaviour in stressed *versus* non-stressed animals, the results of this latter study also raise the possibility of a role for CB_2_ receptors in the co-morbidity of depression and alcohol abuse. Recently it has been shown that transgenic mice engineered to over-express the CB_2_ receptor are resistant to CMS-induced reductions in sucrose consumption and increases in tail suspension test immobility time [138]. The CB_2_ over-expressing mice were also resistant to CMS-induced reductions in brain derived neurotrophic factor (BDNF) levels in the hippocampus. While these results suggest that the CB_2_ over-expression results in a depression-resistant endophenotype, the same study also reported that pharmacological blockade of CB_2_, with chronic administration of the CB_2_ receptor antagonist AM630 for 4 weeks, prevented the effects of CMS on tail suspension test, sucrose intake, CB_2_ receptor gene, BDNF gene and protein expression in wildtype mice. The authors speculate that the chronic antagonist treatment may have lead to increased expression of CB_2_ in key brain regions, thereby mimicking the phenotype of the CB_2_ over-expressing transgenic mice. Taken together, these results indicate that CB_2_ receptors may play an important role in mediating behavioural and molecular effects associated with CMS. 

Early life stress has been linked with a predisposition to psychiatric disorders in later life, resulting in the development of several preclinical models based on this association. One such model is the maternal deprivation (MD) model which involves separation of neonatal rats from the dam for a single 24 hour episode resulting in long-lasting behavioural, neurochemical and immune changes and has been proposed as a model of several neuropsychiatric disorders including depression. The depressive-like phenotype associated with MD includes decreased latency to immobility in the forced swim test [177,178], reduced locomotor activity and social investigatory behaviour [178] and enhanced impulsivity [179]. Alterations in the endocannabinoid system have been demonstrated in this model where MD is associated with enhanced hippocampal 2-AG levels [180] and reduced CB_1_ and increased CB_2_ receptor expression in the hippocampus [70]. Although both male and female MD rats exhibited a comparable increase in hippocampal CB_2_ receptor expression, CB_1_ receptor expression demonstrated sexual dimorphism, with a greater MD-related decrease observed in males when compared to females [70]. Examination of gender-specific effects is of particularly importance due to the enhanced prevalence of neurodevelopmental and psychiatric disorders in women. Detailed examination of the location of these receptors demonstrates that hippocampal CB_2_ receptors are located on dendritic terminals and not microglia [70]. This is consistent with the finding that CB_2_ receptors are located post-synaptically in the hippocampus [55] in comparison to the CB_1_ receptor that is primarily located on pre-synaptic GABAergic and glutamatergic terminals [181,182]. It remains to be determined if similar alterations in the expression of cannabinoid receptors occurs in other brain regions in this model. Thus, MD-induced changes in CB_2_ receptor expression, and other components of the endocannabinoid system may underlie some of the behavioural, cognitive and neuroendocrine changes observed in this model and in the neuropsychiatric disorders it models.

**Figure 1:**
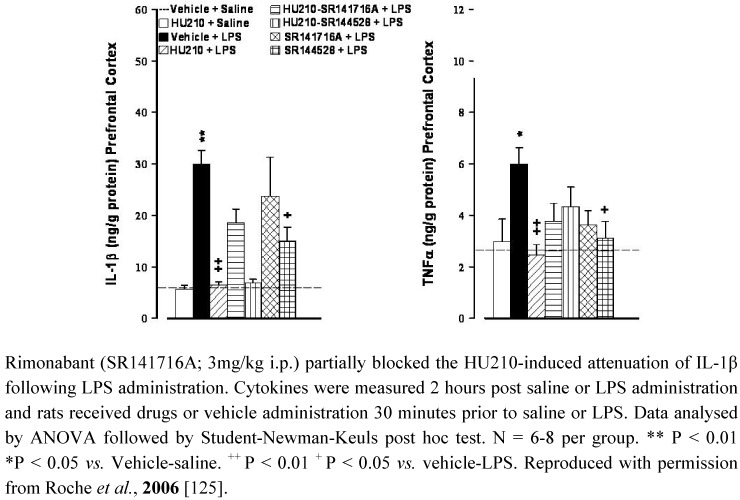
The CB_1_/CB_2_ receptor agonist HU210 (100 µg/kg i.p.) and CB_2_ receptor antagonist SR144528 (3mg/kg i.p.) attenuated LPS (100 µg/kg i.p)-induced increases in IL-1β and TNFα levels in the prefrontal cortex in rats.

**Figure 2:**
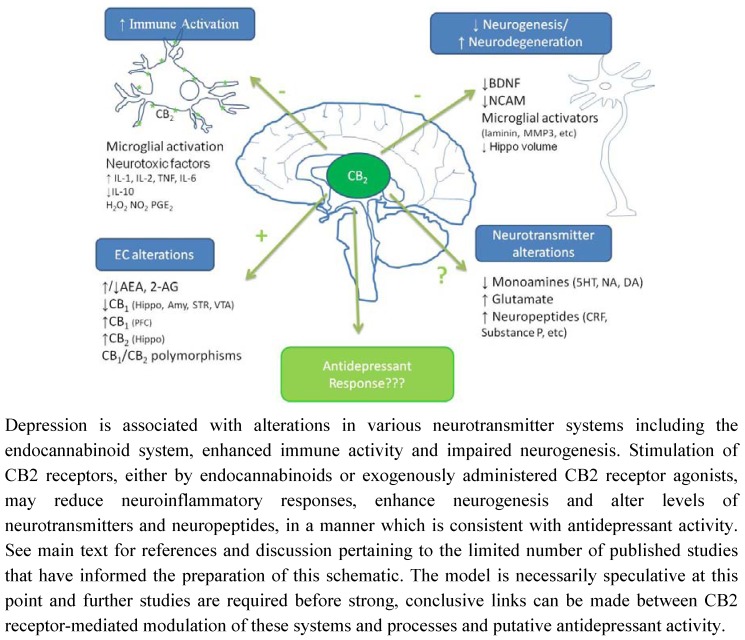
Putative mechanisms underpinning potential antidepressant effect of CB_2_ receptor stimulation.

The functional significance of the alterations in cannabinoid receptor expression in animal models of depression remains to be determined however, as previously highlighted, CB_1_ and CB_2_ receptors modulate neural stem cell proliferation in culture [26,183]) and/or in adult mice [25,129,184,185]. Impaired hippocampal neurogenesis has been proposed to underlie the pathophysiology of depression (for reviews see [186,187,188]). Neurogenesis relies on several factors including neurotrophins such as brain derived neurotrophic factor (BDNF), a reduction in which has been observed in depressed patients [189,190,191] and in preclinical models including CMS [192] and MD [193]. Antidepressants, as a class, reverse pathological stress-induced reductions in adult hippocampal neurogenesis [194], an effect at least partially mediated by enhancing BDNF, TrkB receptor signaling and activation of MAPK/ERK pathways [195,196,197,198]. Similarly, chronic, but not acute, treatment of rats with the CB_1_/CB_2_ agonist HU210, but not the selective CB_1_ agonist AM281, enhanced hippocampal neurogenesis and elicited antidepressant-like behavioural effects [131]. The inability of AM281 to modulate neurogenesis or immobility behaviour may indicate a role for CB_2_ receptors in the effects of HU210, promoting neuronal survival and differentiation in the hippocampus. In addition, WIN55,212-2 induced activation of both CB_1_ and CB_2_ receptors enhances neurogenesis in the hippocampus of aged rats [132]. As highlighted earlier, immunological mediators also modulate neurogenesis, with high levels of pro-inflammatory cytokines considered detrimental to neuronal viability. It has been proposed that the reduced neurogenesis observed in depression is due, at least in part, to the enhanced immune activation and elevated cytokine levels that are a feature of this psychiatric disorder [199], an effect also demonstrated in several animal models including CMS and MD [178,200,201]. Furthermore, immune stimuli such as endotoxins activate microglia and enhance inflammatory cytokine levels in the brain resulting in reduced BDNF [202], reduced neurogenesis [203,204] and depressive-like symptoms [205]. We have previously shown that HU210 attenuates endotoxin-induced increases in IL-1β and TNFα in rat brain, effects partially mediated by CB_1,_ but not CB_2,_ receptors [125]. However, blockade of either CB_1_ or CB_2_ receptors, using rimonabant or SR144528 respectively, also attenuated lipopolysaccaride (LPS)-induced cytokine levels in the brain [125] (Figure 1). In addition, administration of the endotoxin LPS enhances endocannabinoid levels [206,207,208] and increases CB_2_ receptor protein expression in the brain (detected using western immunoblotting) [86]. Based on the clinical and preclinical evidence, we hypothesise that depression is associated with altered endocannabinoid function including that of the brain CB_2_ receptors, activation of which would reduce inflammatory responses, enhance neurogenesis and result in antidepressant activity (Figure 2).

### 5.3. Schizophrenia

The role of the endocannabinoid system in schizophrenia has received considerable attention and has be covered in detail in several recent reviews [209,210] including those within this special issue [211,212]. Therefore, this section will concentrate primarily on the putative role of the CB_2_ receptor in this disorder. 

Ishiguro and colleagues have recently demonstrated that Japanese schizophrenic patients exhibit an increase in the frequency of two single nucleotide polymorphisms (SNPs) in the CB_2_ receptor gene, namely rs12744386 and Q63R, which confer lower functioning of the CB_2_ receptor [160]. Low levels of CB_2_ receptor mRNA and protein in the brain and lympohocytes were associated with the C allele of rs1274486 gene, a genotype commonly observed in schizophrenic patients.

As mentioned earlier, the other SNP (rs2501432) is a mismatch Q63R, the presence of which leads to poor response to CB_2_ ligands [160]. Thus, the presence of both SNPs may synergistically confer enhanced susceptibility to schizophrenia. In line with these observations, schizophrenia is associated with enhanced cerebrospinal fluid levels of anandamide and PEA, and remission from which is associated with reduced anandamide levels and peripheral blood mononuclear cell CB_2_ receptor mRNA expression [213,214,215]. It is unknown if similar changes in CB_2_ receptor expression are observed in the brain, however, neuroleptic treatment reduces G-protein functioning in schizophrenic patients [216], and as such may induce similar effects on G-protein coupled cannabinoid receptors. 

The effect of CB_2_ receptor antagonists on MK-801- or methamphetamine-induced disturbances in pre-pulse inhibition (PPI) have also been examined. PPI is a widely used behavioural test of sensorimotor gating, deficits in which are commonly observed in schizophrenic patients [217,218]. Essentially, PPI refers to the ability of a weak pre-stimulus, a pre-pulse, to inhibit the startle reflex elicited by a subsequent intense stimulus. Administration of the CB_2_ receptor antagonist, AM630, alone, did not alter PPI, however AM630 did augment the MK-801- and metamphetamine-induced reduction in PPI and increase in locomotor activity [160]. It has been proposed that that a decrease in CB_2_ receptor functioning alone does not lead to the development of schizophrenia but that in the presence of other risk factors, reduced CB_2_ receptor functioning may confer enhanced susceptibility to the development of this disorder. 

Disorganised stereotypic behaviour is common in psychotic individuals, mediated by hyperdopaminergic functioning and alleviated by antipsychotic treatment. CB_1_ receptor desensitization due to chronic cannabinoid treatment or CB_1_ receptor antagonism exacerbates dopamine receptor-induced stereotypic behaviours [219,220]. Although the role of CB_2_ receptors in directly mediating or modulating dopamine-induced effects has not been investigated, CB_2_ receptor agonists reduce stereotypic behaviour in a dose-dependent and gender specific manner [60] as outlined in previous sections. In addition, CB_2_ receptors modulate key neurotransmitter systems involved in schizophrenia such as dopaminergic and glutamatergic function, possibly via microglial inhibition. For example, CB_2_ receptor agonists prevent 6-hydroxydopamine induced dopamine depletion [221] and glutamate receptor (AMPA, kainate and NMDA) mediated excitotoxicity [25,79,122,222]. Alterations in cannabinoid receptor expression may induce profound alterations in neurotransmission (dopaminergic and/or glutamatergic) and/or, modulation of hippocampal axonal growth and plasticity, which may confer a predisposition to the development of schizophrenia. MD and/or exposure to cannabinoids during critical neurodevelopmental periods have been proposed to induce such neurochemical alterations which may underlie the psychotic-like behavioural alterations. In addition to the depressive-like phenotype exhibited following MD, this model also results in long-term behavioural alterations that resemble symptoms observed in schizophrenia, including deficits in PPI, latent inhibition and auditory sensory gating [223,224]. The behavioural alterations associated with MD, combined with the neuronal, endocrine and immune alterations observed, support its usefulness and relevance as a model based on the neurodevelopmental hypothesis of schizophrenia. As previously mentioned MD is associated with alterations in the endocannabinoid signalling system including reduced CB_1_ expression and enhanced CB_2_ receptor expression in the hippocampus [70,146,180]. Although an increase in CB_2_ receptor expression in the hippocampus of MD rats may seem at odds with data suggesting that schizophrenia is associated with reduced CB_2_ receptor functioning, it should be noted that although the density of CB_2_ receptors is enhanced, significant impairment in function may exist. In addition, CB_2_ receptor expression was assessed in pre-pubertal MD rats and the density and distribution pattern of this receptor may be different in adults. 

Schizophrenia is associated with altered neuroimmune functioning, primarily an imbalance between type-1 and type-2 immune responses, which is thought to underlie altered neuronal function including neurotransmitter alterations and reduced neurogenesis (for review see [225,226,227,228]). In addition, epidemiological data have demonstrated an association between prenatal infection, enhanced pro-inflammatory cytokine levels and increased risk of psychiatric disorders in later life, including schizophrenia [229], an association exploited in order to develop more ethologically valid models of schizophrenia. This imbalance in immune function is also observed in neurodevelopmental models such as MD [178,200]. Antipsychotic medication and anti-inflammatory agents such as COX-2 inhibitors alleviate psychotic symptoms, correct the imbalance in type-1 and type-2 responses and reduce pro-inflammatory cytokine release [225,226]. It remains to be determined whether the anti-inflammatory effects of CB_2_ receptor agonism might confer protection and enhancement of neuronal function of sufficient magnitude to alleviate psychotic symptoms in schizophrenia.

The enhanced prevalence of schizophrenia and other psychiatric disorders such as anxiety and depression in women highlights the need for more studies examining gender differences in the development of these disorders. Gonadal hormones have been demonstrated to alter expression of cannabinoid receptors [230] and females in general are more sensitive than males to the behavioural effects of cannabinoids [231]. As highlighted in earlier sections, few studies have examined the effect of CB_2_ ligands in animal models of neuropsychiatric disorder and for the most part these studies have been confined to examining effects in male animals. However, it has been demonstrated that the CB_2_ receptor agonist JWH-015 reduces stereotypic behaviour in female mice at a dose (10 mg/kg) that is ineffective in male counterparts [60]. In addition, sexually dimorphic effects on hippocampal CB_1_ receptor expression have been demonstrated in the MD neurodevelopmental animal model, with a more marked MD-related decrease observed in male rats compared with female rats [70]. In comparison, comparable increases in CB_2_ receptor expression were observed in male and female rats. As such further studies are required in order to determine if gender-specific alterations in endocannabinoid function may underlie the development of, and differential susceptibility to, neuropsychiatric disorders. 

## 6. Conclusions and Future Directions

Due to the increased availability of tools which have allowed for a more specific analysis of the neuroanatomical distribution, neurophysiology and neuropharmacology of the CB_2_ receptor, considerable evidence now exists to support the presence of CB_2_ receptors on microglia and subpopulations of neurons within the brain, contrary to the belief widely held previously that this receptor was restricted to peripheral locations. Further studies are required in order to elucidate the role of this receptor under non-pathological conditions, however, central endocannabinoids may act primarily at CB_1_ receptors with possible co-operative effects at CB_2_ receptors, in order to maintain normal homeostasis. In comparison, several pathophysiological conditions are associated with altered CB_1_ and CB_2_ receptor expression and/or function, and under such conditions, endocannabinoid activity at CB_2_ receptors may have greater significance. Such a mechanism would explain why CB_2_ receptor ligands elicit little or no psychoactive effects in experimental settings mimicking normal acute physiological responding, but may exert potent effects in models of disease states such as neurodegenerative disorders and chronic pain. This may have important therapeutic implications as CB_2_ receptor agonists may be devoid of psychoactivity under normal non-pathological conditions and in disorders associated with increased CB_2_ receptor expression in peripheral tissues. However, in conditions associated with increased CB_2_ receptor expression in the brain, these receptors may elicit appreciable CNS effects. For example, increased central CB_2_ receptor expression on microglia as observed in neuroinflammatory and neurodegenerative disorders may be targeted to induce an anti-inflammatory environment that promotes neuroprotection and/or neuroregeneration. Similarly, CB_2_ receptors on microglia and neurons in critical brain regions involved in regulating emotion may be altered in psychiatric disorders such as anxiety, depression and schizophrenia, and targeting these receptors may reduce neuroinflammatory processes, enhance neurogenesis and modulate neurotransmitter systems thereby alleviating symptoms associated with these disorders (Figure 2).

More studies evaluating the involvement of CB_2_ receptors in psychiatric disorders are both justified and required. Such studies should employ the full range of tools that are available to study the CB_2_ receptor, including reliable, well-characterised and validated antibodies and primers, selective CB_2_ receptor agonists and antagonists and CB_2_ receptor transgenic mice (knockouts or overexpressing) to evaluate the interaction between these receptors and other components of the endocannabinoid system in appropriate animal models. The selectivity of CB_2_ receptor ligands continues to improve with several compounds entering clinical trials for non-psychiatric disorders (e.g., osteoarthritis, dental pain). Careful evaluation of the side effects associated with chronic treatment of these agents will provide further insight into the potential role of CB_2_ in regulating neurophysiological function. In addition, the development of more selective probes such as high quality antibodies, and studies which employ a variety of anatomical, functional and biochemical techniques in order to evaluate the expression and function of CB_2_ receptors will lead to increased knowledge on the role of this receptor in the brain. As we have seen, some very interesting data have begun to emerge. However, the evidence to-date for a role of CB_2_ receptors in neuropsychiatric disorders is largely indirect, and so further studies are required to determine the precise pathophysiological contribution of the CB_2_ receptor and its true potential as a viable therapeutic target in neuropsychiatric disease. 

## Abbreviations

2-AG: 2-arachidonyl glycerolc
AEA: anandamide
AMPA: α-amino-3-hydroxy-5-methyl-4-isoxazole propionic acid receptor
BDNF: brain derived neurotrophic factor
CMS: chronic mild stress
CNS: central nervous system
EAE: experimental auto- immune encephalomyelitis
ERK: extracellular signal-regulated kinase
FAAH: fatty acid amide hydrolyase
IL-1: interleukin-1
JNK: Jun *N*-terminal protein kinase
MAPK: mitogen-activated protein kinase
MD: maternal deprivation
MAGL: monoacylglycerol lipase
NMDA: *N*-methyl-D-aspartic acid
OEA: *N*-oleoylethanolamide
PEA: Palmitoylethanolamide
PI3K: Phosphoinositide 3-kinase
PPAR: peroxisome proliferator- activated receptors
PPI: pre-pulse inhibition
TNFα: tumour necrosis factor-α
TRPV1: transient receptor potential vanilloid 1

## Endogenous Ligands and Pharmacological Agents Cited

Endocannabinoids: 2-AG, AEA

Fatty acid amides: PEA, OEA

Non-selective cannabinoid receptor agonists: HU210, WIN55,212-2

CB_1_ receptor agonists: AM281

CB_1_ receptor antagonists/inverse agonists: Rimonabant (SR141617A), AM251

CB_2_ receptor agonists: JWH-015, JWH-133, AM1241, GW405833, PEA (partial)

CB_2_ receptor antagonists: AM630, SR144528, JTE-907
